# The Virulence of *Metarhizium rileyi* to *Locusta migratoria* Is Determined by the Ability of the Fungus to Respond to Carbon and Nitrogen Sources

**DOI:** 10.3390/ijms26094156

**Published:** 2025-04-27

**Authors:** Yunhao Yao, Mei Li, Qingqing Liu, Qiuyue Huang, Shuo Yang, Bin Chen, Yuejin Peng

**Affiliations:** College of Plant Protection, Yunnan Agricultural University, Kunming 650201, China; yaoyunh@163.com (Y.Y.); 15187736091@163.com (M.L.); lq01067@163.com (Q.L.); h13896809805@163.com (Q.H.); 13988308817@139.com (S.Y.)

**Keywords:** *Metarhizium rileyi*, *Locusta migratoria*, carbon and nitrogen metabolism, biological control, virulence

## Abstract

Insects are among the most diverse and abundant organisms on Earth, and their population dynamics are strongly influenced by entomopathogenic fungi. This study examines the role of carbon and nitrogen metabolism in the virulence of the entomopathogenic fungus *Metarhizium rileyi* against the migratory locust, *Locusta migratoria*. The findings demonstrate that the capacity of *M. rileyi* to utilize different carbon and nitrogen sources is a key factor in its virulence. Specifically, two strains of *M. rileyi* (PPDB201006 and SZCY201010) exhibited distinct metabolic abilities, with PPDB201006 displaying superior growth and enzyme activities on various carbon and nitrogen sources compared to SZCY201010. These metabolic differences were associated with significant variations in virulence, as PPDB201006 induced higher mortality rates in *L. migratoria* than SZCY201010. Metabolomics analysis revealed that infection by *M. rileyi* led to substantial alterations in the hemolymph metabolites of *L. migratoria*, particularly in organic acids, amino acids, sugars, and lipids. These results emphasize the significance of carbon and nitrogen metabolism in the pathogenicity of entomopathogenic fungi and offer new perspectives for optimizing their application as biological control agents. This study not only improves our understanding of fungal virulence mechanisms but also contributes to the development of more effective and sustainable pest management strategies.

## 1. Introduction

*L. migratoria*, a member of the family Orthopteridae, is a highly social insect and one of the most destructive agricultural pests worldwide due to its rapid reproductive capacity and large population sizes [1]. Unlike many other pest species, *L. migratoria* populations can expand rapidly and have high densities, posing a severe threat to food security and crop production in many regions [2,3]. Despite these challenges, the primary method for controlling *L. migratoria* still relies on chemical pesticides. However, prolonged pesticide use has led to widespread resistance in locust populations, along with adverse effects on the environment, human health, and non-target species [4,5,6]. Consequently, several countries have begun exploring more environmentally friendly and effective alternatives, such as developing new low-toxicity, low-residue pesticides. In China, the proportion of biological control methods has been increasing annually, including the use of biopesticides such as locust microsporidium and *Metarhizium* species for population management [7,8].

Entomopathogenic fungi play a crucial role in regulating insect populations in natural ecosystems [9]. Due to their high insecticidal efficacy and environmental safety, these fungi are regarded as promising alternatives to chemical pesticides [10]. The infection process of entomopathogenic fungi involves multiple stages: host recognition, conidial attachment to the insect cuticle, germination and bud formation, penetration into the host’s cuticle, and eventual colonization of the hemocoel [11]. This invasion is influenced by various factors, with the insect’s cuticle serving as the primary barrier [12]. During penetration, fungal pathogens rely on hydrolytic enzymes such as chitinases and lipases to break down the structural components of the insect cuticle, enabling entry into the hemolymph. Once inside, the fungi compromise the host’s immune defenses, utilize host-derived nutrients for reproduction, and ultimately cause host mortality [13]. Several entomopathogenic fungi, including *Metarhizium anisopliae* [9], *Metarhizium acridum* [14], and *Beauveria bassiana* [15], have demonstrated significant efficacy in controlling locust populations. *M. rileyi*, also known as *Nomuraea rileyi*, is a filamentous fungus widely recognized for its effectiveness in controlling lepidopteran pests in the field [16]. It has been reported to successfully manage pests such as the armyworm [16]. However, there are no documented studies on the use of *M. rileyi* for locust control.

The mechanisms of carbon and nitrogen metabolism in pathogenic fungi are known to directly influence their growth, reproduction, and pathogenicity. During infection, entomopathogenic fungi secrete various metabolites that can impact insect physiology. For instance, toxins and enzymes produced by *M. anisopliae* can compromise the physical barrier of the insect gut while disrupting the metabolic balance of gut microbiota [16]. At the same time, pathogenic fungi undergo fine-tuned regulation of multiple metabolic networks within the host environment, involving transcription factors and signaling pathways [17]. For example, MaNmrA, a nitrogen metabolism repressor in *M. acridum*, plays a key role in regulating carbon and nitrogen source utilization. Deletion of the *MaNmrA* gene has been shown to affect growth rate, sporulation, and the ability to metabolize carbon and nitrogen sources in *M. acridum* [18]. Similarly, the transcription factor Mavib-1 in *M. anisopliae* negatively regulates conidiation by regulating carbon and nitrogen metabolism. Studies indicate that Mavib-1 knockdown accelerates conidial formation, whereas its overexpression delays conidiation, particularly under carbon- or nitrogen-limited conditions [19]. In *B. bassiana*, BbAreA positively regulates nitrogen metabolism and hyperosmotic stress tolerance while negatively influencing oxidative and hypoxic stress adaptation [20]. The metabolic processes of *B. bassiana*, especially lipid metabolism (*BbYap1* [21], *BbOle1* [22] and *MARVEL* family genes [23]) and iron metabolism (*CFEM* family genes [24,25] and transcription factor *BbHapX* [26]), play an important role in its virulence. However, different entomopathogenic fungi adopt distinct strategies for responding to and utilizing carbon and nitrogen sources when infecting different insect hosts [20]. The mechanism by which *M. rileyi* recognizes and metabolizes host-derived carbon and nitrogen sources during locust infection remains unknown.

To investigate the differences in virulence between *M. rileyi* strains SZCY201010 and PPDB201006 against *L. migratoria*, this study employed non-targeted metabolomics analysis. The intestinal metabolites of *L. migratoria* infected with the two fungal strains were examined, revealing that the fungus’ response to carbon and nitrogen metabolism plays a critical role in determining differences in its virulence. This study not only identifies a new microbial resource for the biological control of orthopteran pests but also provides new insights into the mechanisms by which entomopathogenic fungi regulate pest populations.

## 2. Results

### 2.1. The Pathogenic Fungi PPDB201006 and SZCY201010 Were Identified as M. rileyi

**Figure 1:**
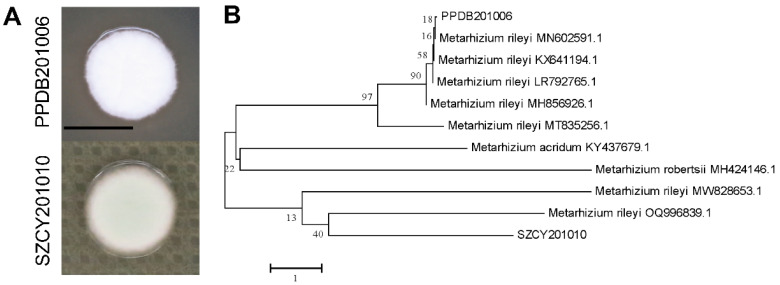
Isolation and identification of *M. rileyi* PPDB201006 and SZCY201010: (**A**) Colony morphology of strains PPDB201006 and SZCY201010 on SMAY plates collected from Baoshan and Shizong in Yunnan Province, China. (**B**) Phylogenetic analysis of strains PPDB201006 and SZCY201010 based on ITS sequences. In the phylogenetic tree constructed using the neighbor-joining method, branches with bootstrap support values greater than 50% are labeled based on 1000 bootstrap replicates.

Colony morphology analysis revealed that strain PPDB201006 exhibited a significantly faster growth rate compared to strain SZCY201010 (Figure 1A). ITS sequence alignment and phylogenetic analysis confirmed that both strains (PPDB201006 and SZCY201010) were closely related to *M. rileyi*. Despite belonging to the same species, a considerable genetic distance was observed between the two strains (Figure 1B and Figure S1).

### 2.2. Virulence of L. migratoria Infected by M. rileyi PPDB201006 and SZCY201010 by Different Methods

**Figure 2:**
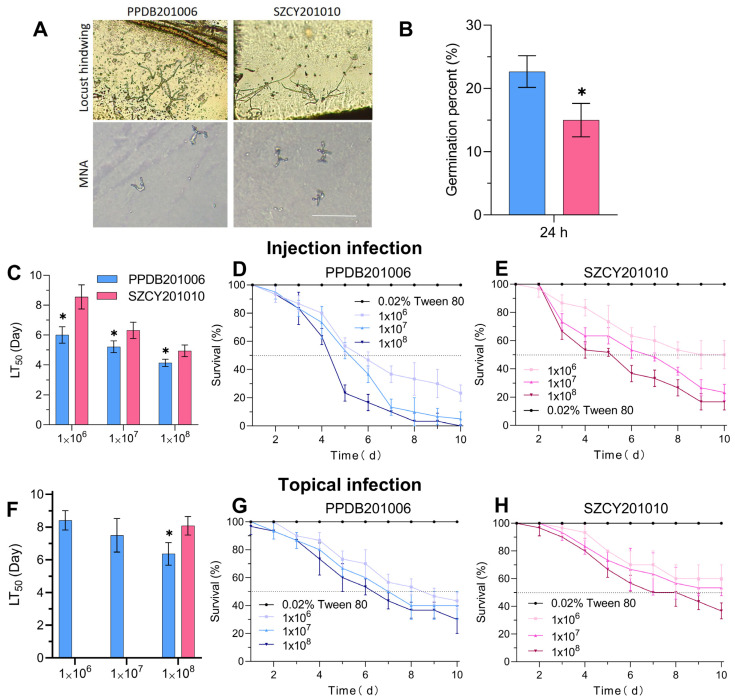
Assessment of *L. migratoria* virulence following infection by *M. rileyi* PPDB201006 and SZCY201010 via different methods: (**A**,**B**) Conidial germination of *M. rileyi* PPDB201006 and SZCY201010 on locust hindwings and MNA plates at 24 h. (**C**–**H**) Survival rate and LT_50_ values of *L. migratoria* following injection or topical infection with 0.02% Tween 80 and conidial suspensions of strains PPDB201006 and SZCY201010 at different concentrations. Tukey’s Honestly Significant Difference (HSD) test: *p* < 0.05 (*); error bars indicate standard deviation.

The virulence of *M. rileyi* PPDB201006 and SZCY201010 against *L. migratoria* was evaluated using injection and topical application methods. Spore germination was assessed under nutrient-deficient conditions using locust hindwings and MNA plates (Figure 2A). At 24 h, a significant difference in spore germination rates was observed between the two strains, with PPDB201006 showing a notably higher germination rate than SZCY201010 (Figure 2B). Injection assays revealed distinct differences in *L. migratoria* LT_50_ values at conidial concentrations of 1.0 × 10^6^, 1.0 × 10^7^, and 1.0 × 10^8^ conidia/mL. Compared to PPDB201006, the LT_50_ values for *L. migratoria* infected with SZCY201010 were increased by 2.94, 1.01, and 0.79 days in the respective groups (Figure 2C–E). In contrast, for topical infection, a significant difference in *L. migratoria* LT_50_ values between the two strains was only observed at 1.0 × 10^8^ conidia/mL, where the LT_50_ for SZCY201010 was 0.95 days longer than that of PPDB201006 (Figure 2F–H).

### 2.3. Effects of M. rileyi PPDB201006 and SZCY201010 on Metabolism of L. migratoria

**Figure 3:**
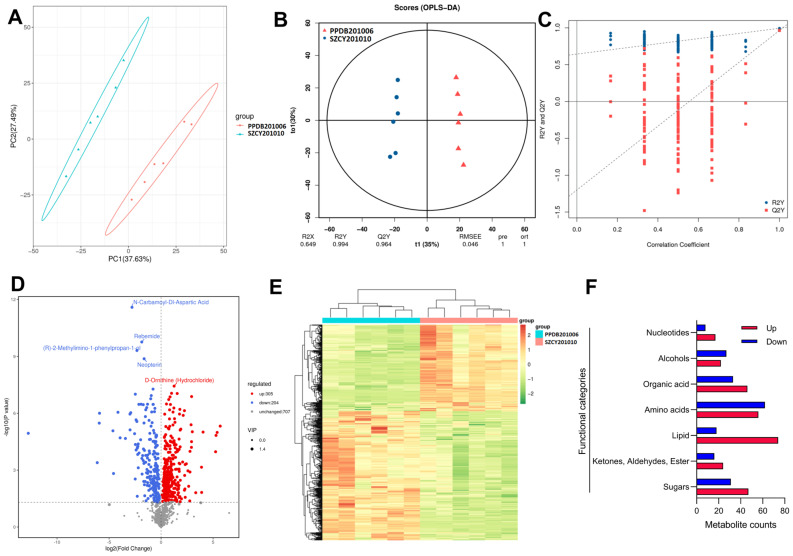
Hemolymph metabolome analysis of *L. migratoria* infected with *M. rileyi* SZCY201010 and PPDB201006: (**A**) PCA score plot of the hemolymph metabolome of *L. migratoria* infected with *M. rileyi* SZCY201010 and PPDB201006; (**B**) OPLS-DA score plot; (**C**) OPLS-DA model permutation test; (**D**) Volcano plot of differential metabolites in *L. migratoria* hemolymph infected with different *M. rileyi* strains; (**E**) clustering heat map of differential metabolites. (**F**) The functional distribution and analysis of metabolomics in the hemolymph of insects.

Principal component analysis (PCA) was used to examine the overall distribution of samples and assess the stability of the analytical process. The PCA results indicated a low degree of dispersion among the infected groups of *M. rileyi* PPDB201006 and SZCY201010, demonstrating that the results were stable and reliable. OPLS-DA was further employed to extract information on intergroup variability more effectively (Figure 3A). The OPLS-DA score plots (Figure 3B) showed clear differences between the PPDB201006 and SZCY201010 infection groups, with model parameters including R2X = 0.649, R2Y = 0.994, and Q2Y = 0.964, indicating that the OPLS-DA model was not overfitted and had a strong interpretability for subsequent analysis. To evaluate the reliability of the OPLS-DA model, a permutation test was conducted by randomly shuffling sample groupings and recalculating R2Y and Q2Y based on the permuted data. The results demonstrated that the slope of the Q2Y fitting regression line was positive, confirming the validity of the model. Additionally, the blue dots were consistently positioned above the red dots, indicating good independence between the training set and the test set in the modeling process (Figure 3C).

To analyze the expression levels of hemolymph metabolites in *L. migratoria manilensis* infected by different strains of *M. rileyi* (SZCY201010 and PPDB201006), a total of 1216 identified metabolites were further examined using volcano plot analysis. Differentially expressed metabolites were screened based on VIP values, with significant metabolites selected using the criteria of a fold change score = 1 and VIP = 1. The screening results are presented in Figure 3D. In the volcano plot, each dot represents a metabolite: blue dots indicate down-regulated differentially expressed metabolites, red dots represent up-regulated differentially expressed metabolites, and gray dots correspond to metabolites that were detected but showed no significant difference. The top five metabolites were labeled based on their *p*-values. Among the down-regulated metabolites, N-Carbamoyl-DL-Aspartic Acid, Rebemide, (R)-2-Methylimino-1-phenylpropan-1-ol, and Neopterin were identified, whereas D-Ornithine (Hydrochloride) was the most significantly up-regulated metabolite. A heat map without substance names was generated through inter-group clustering (Figure 3E). The differential metabolites mainly included nucleotides (17 up-regulated, 8 down-regulated), alcohols (22 up-regulated, 27 down-regulated), organic acids (46 up-regulated, 33 down-regulated), amino acids (56 up-regulated, 8 down-regulated), lipids (74 up-regulated, 18 down-regulated), ketones, esters, and aldehydes (24 up-regulated, 16 down-regulated), and sugars (47 up-regulated, 31 down-regulated) (Figure 3F and Table S1).

**Figure 4:**
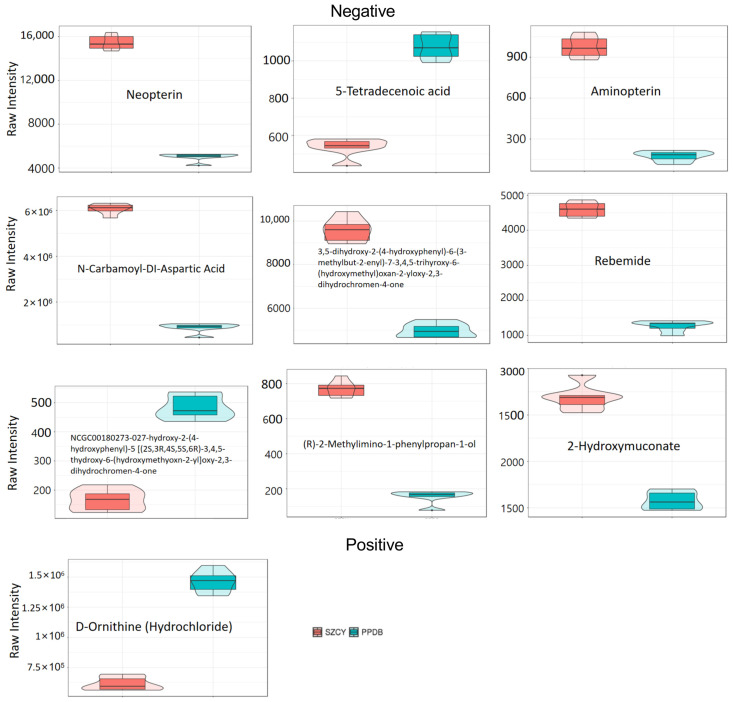
Violin plot illustrating the significantly different metabolite concentrations between *M. rileyi* strains PPDB201006 (green) and SZCY201010 (red).

Each of the selected metabolites is presented in Figure 4. Among them, the most significantly down-regulated metabolite was Aminopterin (FC = 5.56, *p* < 0.01, VIP = 1.68), while the most significantly up-regulated metabolite was D-Ornithine (Hydrochloride) (FC = 0.42, *p* < 0.01, VIP = 1.68).

These findings suggest that *L. migratoria* undergoes metabolic transformation during infection by *M. rileyi*, particularly in organic acids, amino acids, sugars, and lipids. Additionally, the metabolic alteration mechanisms differ between *M. rileyi* strains.

### 2.4. Analysis of Differentially Expressed Metabolite Pathways and Enrichment Analysis

**Figure 5:**
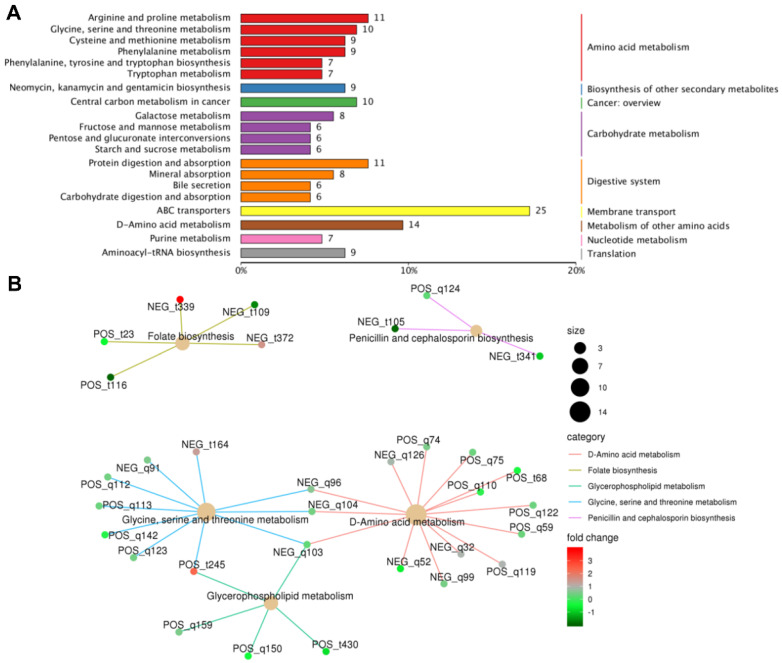
KEGG pathway enrichment analysis of *L. migratoria* following infection with *M. rileyi*: (**A**) Classification map for differentially expressed metabolite pathways in each group. (**B**) KEGG enrichment network of differential metabolites.

A KEGG pathway enrichment analysis of differentially expressed metabolites was conducted using the hypergeometric test method in cluster Profiler. The top 20 pathways with the highest number of annotated differential metabolites were selected, with key pathways primarily involving amino acid metabolism, biosynthesis of secondary metabolites, cancer-related pathways, carbohydrate metabolism, digestive system processes, membrane transport, the metabolism of other amino acids, nucleotide metabolism, and translation pathways (Figure 5A).

Further enrichment analysis of the metabolic network, based on the relationship between metabolites and their associated pathways, revealed several critical biosynthetic and metabolic changes. For example, dihydropteroate (NEG_t339) and dihydropteroate (NEG_T339), along with 4-(β-D-ribofuranosyl)phenol 5′-phosphate, were upregulated in the folate biosynthesis pathway. L-Valine (POS_q124), isopenicillin N (NEG_t105), and deacetoxycephalosporin C (NEG_t341) were downregulated in the penicillin and cephalosporin biosynthesis pathway. Additionally, the upregulation of choline was found to simultaneously influence glycine, serine, and threonine metabolism, as well as glycerophospholipid metabolism. Meanwhile, L-aspartic acid (NEG_q96), L-threonine (NEG_q104), and L-serine (NEG_q103) were found to be involved in multiple metabolic pathways, including glycine, serine, and threonine metabolism, glycerophospholipid metabolism, and D-amino acid metabolism (Figure 5B).

### 2.5. PPDB201006 and SZCY201010 Showed Significant Differences in Response to Carbon Sources and Nitrogen Sources

**Figure 6:**
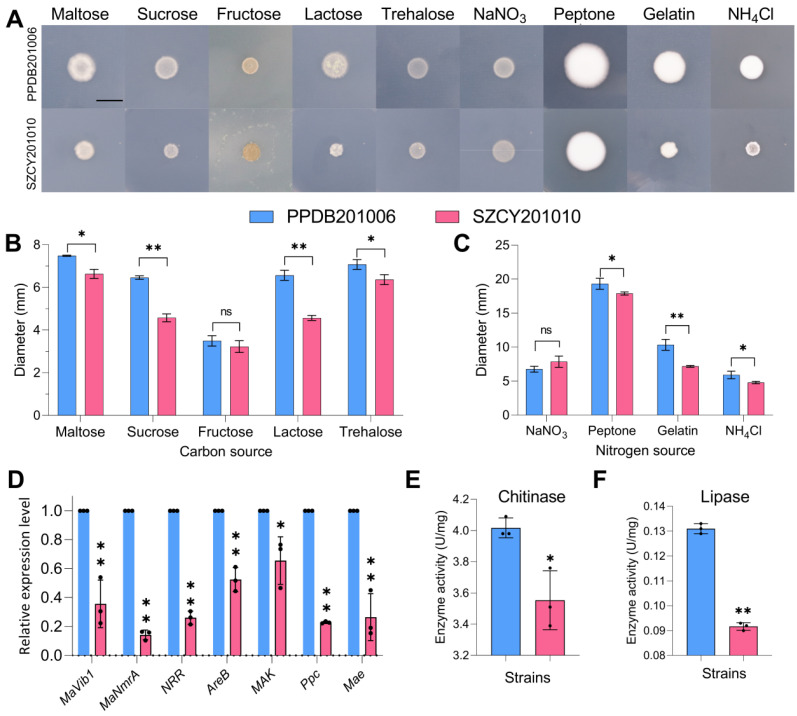
Growth characteristics and carbon and nitrogen metabolism in *M. rileyi* PPDB201006 and SZCY201010: (**A**) differences in fungal colony morphology on plates with specific carbon and nitrogen sources; (**B**) colony diameters on different carbon sources; (**C**) Colony diameters on different nitrogen sources; (**D**) expression levels of genes associated with carbon and nitrogen metabolism in both *M. rileyi* strains; (**E**,**F**) chitinase and lipase activities in PPDB201006 and SZCY201010. Scale: 1 cm. Tukey’s Honestly Significant Difference (HSD) test: *p* < 0.05 (*), *p* < 0.01 (**); error bars indicate standard deviation.

The growth responses of PPDB201006 and SZCY201010 on plates with different carbon and nitrogen sources exhibited distinct colony morphologies (Figure 6A). When maltose, sucrose, lactose, or trehalose was the sole carbon source, the colony diameter of PPDB201006 was significantly larger than that of SZCY201010. However, no significant difference was observed between the two strains when fructose was the sole carbon source (Figure 6B). Similarly, the two strains responded differently to various nitrogen sources. The colony diameter of PPDB201006 was significantly larger than that of SZCY201010 when peptone, gelatin, or NH_4_Cl served as the sole nitrogen source. However, no significant difference was observed between the strains when NaNO_3_ was used as the nitrogen source (Figure 6C). RT-qPCR analysis on the fourth day of culture revealed that the expression levels of key genes involved in carbon and nitrogen metabolism, including *Mavib1*, *MaNmrA*, *NRR*, *AreB*, *MAK*, *Ppc*, and *Mae*, were significantly higher in PPDB201006 than in SZCY201010 (Figure 6D). Additionally, enzyme activity assays demonstrated significant differences in fungal enzyme production between the two strains. The chitinase and lipase activities of PPDB201006 were significantly higher than those of SZCY201010 (Figure 6E,F).

## 3. Discussion

The study of entomopathogenic fungi and their interactions with insect hosts has received increasing attention due to their potential as biological control agents. This research investigates how *M. rileyi* exerts its virulence against the migratory locust, *L. migratoria*, with a particular focus on the role of carbon and nitrogen metabolism. Our findings demonstrate that the ability of *M. rileyi* to utilize different carbon and nitrogen sources is a key factor in determining its virulence.

In recent years, the application of biological control strategies for locust management has been on the rise. Several field trials have demonstrated that the effectiveness of pathogenic fungi can be significantly improved when they are used in combination with synergists. These studies provide strong data supporting the practical implementation of such approaches. For instance, phenidone combined with *B*. *bassiana* significantly increased the mortality of migratory locusts [27]. The well-timed co-application of *Paranosema locustae* and *B. bassiana* was shown to be more effective than either treatment alone [28]. Additionally, *B. bassiana* combined with the insect growth regulators diflubenzuron and novaluron exhibited a synergistic effect against migratory locusts [29]. These findings emphasize the potential to improve the efficacy of entomopathogenic fungi in locust control. In our study, *M. rileyi* was identified as a highly virulent pathogen of *L. migratoria*. A comparative analysis of two *M. rileyi* strains, PPDB201006 and SZCY201010, revealed that PPDB201006 consistently exhibited a higher virulence, as indicated by its lower LT_50_ values in both injection and topical infection assays. This increased virulence is likely attributable to PPDB201006’s superior metabolic capabilities and enzyme activities, which increase its ability to overcome host defenses and exploit available nutrients.

Our findings align with previous studies that have highlighted the role of carbon and nitrogen metabolism in the virulence of entomopathogenic fungi. For example, the nitrogen catabolite repression regulator AreA in *B. bassiana* has been shown to balance fungal nutrient utilization and virulence [20]. Similarly, the transcription factor Mavib-1 in *M. acridum* negatively regulates conidiation by affecting carbon and nitrogen metabolism [19]. Furthermore, in *Magnaporthe oryzae*, *Nut1*, as a global nitrogen regulatory gene, can regulate the expression of nitrogen metabolism utilization and the pathogenic gene *MPG1* under nitrogen source restriction conditions [30]. In *Fusarium oxysporum*, *Fnr1* can mediate the adaptation of the bacteria to nitrogen-limiting conditions by regulating the acquisition and utilization of secondary nitrogen metabolites [31]. These studies, together with our results, emphasize the crucial role of nutrient metabolism in shaping the pathogenicity of filamentous pathogenic fungi.

Carbon and nitrogen are essential nutrients for fungal growth and metabolism. In entomopathogenic fungi, their availability and utilization within the insect host play a crucial role in the infection process and ultimately determine fungal virulence. Our findings indicate that the two *M. rileyi* strains exhibit distinct capabilities to utilize carbon and nitrogen sources, which correlate with their varying levels of virulence against *L. migratoria*.

The growth phenotype analysis carried out on different carbon and nitrogen sources revealed significant metabolic differences between the two strains. PPDB201006 demonstrated superior growth on multiple carbon sources, including maltose, sucrose, lactose, and trehalose, compared to SZCY201010. Similarly, under nitrogen-limited conditions, PPDB201006 exhibited improved growth on peptone, gelatin, and NH_4_Cl. These findings suggest that PPDB201006’s ability to metabolize a broader range of carbon and nitrogen sources may contribute to its higher virulence. This is further supported by the upregulated expression of key genes involved in carbon and nitrogen metabolism, such as *Mavib-1* and *MaNmrA*, which were significantly higher in PPDB201006 than in SZCY201010.

Previous studies have examined the effects of chlorantraniliprole and *M. anisopliae* on the activity of detoxification and immune-related enzymes, such as glutathione-S transferase (GST), esterases (ESTs), phenoloxidase (PO), and chitinase (CHI), in migratory locusts [32]. In our study, differential gene expression likely influenced the production of enzymes critical for fungal pathogenesis, such as chitinase and lipase. PPDB201006 exhibited significantly higher chitinase and lipase activities than SZCY201010. These enzymes play a vital role in degrading the insect cuticle, facilitating fungal penetration, and enhancing virulence. The ability to efficiently break down the insect cuticle and utilize the released nutrients is a key factor contributing to the success of entomopathogenic fungi.

Hemolymph, the circulating body fluid in insects, functions similarly to blood in higher animals and plays a crucial role in various physiological processes. The insect hemolymph contains immune cells, such as hemocytes, and fat bodies that synthesize and secrete antimicrobial peptides capable of directly eliminating invading pathogens [33]. Additionally, the metabolites in the hemolymph, including glucose, amino acids, and fatty acids, serve as energy sources for immune responses [34]. Moreover, signaling molecules such as nitric oxide and reactive oxygen species regulate immune cell activity and regulate immune response intensity [35]. In our study, a non-targeted metabolomics analysis revealed significant differences in metabolic profiles. The most notable changes were observed in organic acids, amino acids, sugars, and lipids, which are key components in the insect’s immune defense and overall physiology. These distinct metabolic shifts suggest that the two fungal strains trigger different physiological responses in the host, potentially affecting the outcome of the infection. For example, the upregulation of specific amino acids and organic acids in insects infected with PPDB201006 may indicate the strain’s ability to alter host metabolic pathways to its advantage.

The ecological and agricultural implications of these findings are substantial. Entomopathogenic fungi are increasingly being employed as biological control agents due to their high efficacy and environmental compatibility. A deeper understanding of how these fungi exploit carbon and nitrogen metabolism to increase virulence can aid in optimizing their use in pest management strategies. For instance, manipulating the nutritional environment of the insect host or selecting fungal strains with superior metabolic adaptability could improve the effectiveness of biological control measures.

In our research, the example of *M. rileyi* controlling Orthoptera pests was reported first, which effectively expanded the biological control application of *M. rileyi*. Identifying the regulatory networks that govern gene expression related to nutrient utilization and enzyme production could provide deeper insights into the mechanisms underlying fungal pathogenicity. Additionally, investigating the potential of genetic engineering to enhance the metabolic capacity of entomopathogenic fungi could contribute to the development of more effective biological control agents. However, due to the specific differences in the *M. rileyi* strain, such as a long culture period and limited sporidium production, it may be difficult to extend our results to a wider range of biological control applications. Field studies are also crucial for validating laboratory findings and evaluating the practical applicability of entomopathogenic fungi in pest management. Environmental factors (Such as humidity, light and temperature), insect population dynamics, and interactions with other pests and pathogens can all influence the effectiveness of these fungi in natural settings. Therefore, extensive field trials are necessary to refine their application and optimize their use in real-world pest control strategies.

## 4. Materials and Methods

### 4.1. Strains, Medium and Insect

The *M. rileyi* strains SZCY201010 and PPDB201006 used in this study were isolated from *Spodoptera frugiperda* and preserved at the State Key Laboratory of Conservation and Utilization of Biological Resources in Yunnan, China [36]. Before the experiments, conidia of *M. rileyi* were collected after nine days of growth on Sabouraud Maltose Agar with Yeast Extract (SMAY) medium (1% yeast extract, 4% maltose, 1% peptone, and 1.5% agar). Mycelia were removed by filtration through sterile lens paper. *M. rileyi* conidial suspensions at concentrations of 1 × 10^6^ conidia/mL, 1 × 10^7^ conidia/mL, and 1 × 10^8^ conidia/mL were prepared using a hemocytometer under a microscope with 0.02% Tween 80. The *L. migratoria* nymphs used for virulence assays were third-instar individuals reared under laboratory of the School of Plant Protection, Yunnan Agricultural University, in a greenhouse. Fungal molecular identification was conducted using ITS1 and ITS4 sequences, with sequence alignment performed using the ClustalX function in MEGA 7.0. Phylogenetic analysis was carried out using the neighbor-joining method [37].

### 4.2. Determination of Fungal Growth Phenotype

Referring to the previous research methods [38], we, respectively, determined the ability of fungi to respond to nutrients by using the culture medium containing a single carbon source and a nitrogen source. A 1 × 10^7^ conidia/mL suspension of *M. rileyi* was applied at 1 µL per spot onto various phenotyping plates and incubated for seven days at 25 °C under a 12 h photoperiod. Colony diameters were measured twice per dish, and images were captured as needed. Each experiment was conducted in triplicate, with three parallel controls. The fungal growth phenotype was assessed using different carbon and nitrogen sources. Carbon sources included 3% fructose, 3% sucrose, 3% trehalose, 3% lactose, and 3% maltose, while nitrogen sources included 0.3% NH_4_Cl, 0.3% gelatin, 0.3% peptone, and 0.3% NaNO_3_. Czapek-Dox medium (CZA: 3% sucrose, 0.1% K_2_HPO_4_, 0.05% KCl, 0.3% NaNO_3_, 0.05% MgSO_4_, 0.001% FeSO_4_, 1.5% agar) was used as a chemically defined medium.

### 4.3. Phenotypic Determination of Conidial Germination

To simulate the germination rate of *M. rileyi* spores under the nutrient-poor conditions of an insect’s body surface, 100 µL of a conidial suspension at a concentration of 1 × 10^7^ conidia/mL was evenly spread onto plates containing 1.5% agarose. The plates were incubated at 25 °C for 24 h, after which the germination rate was assessed under a microscope. Three different areas were selected for each sample, and the experiment was repeated three times.

To further simulate conidial germination and infestation on the insect body, *L. migratoria* wings were used as test materials. The wings were first treated with 10% NaClO solution, rinsed four times with sterile water, and then flattened onto slides. The conidial suspension of the test strain was evenly applied to the wing surface and incubated at 25 °C for 24 h. The samples were then observed and photographed under a microscope. Each experiment was repeated three times.

### 4.4. Virulence Test

Each locust was treated with a conidial suspension at concentrations of 1 × 10^6^, 1 × 10^7^, and 1 × 10^8^ conidia/mL. The insects were either immersed in the suspension for 10 s or injected with 5 μL. Each treatment group contained 30 insects, with three replicates per group. Corn leaves were provided daily as food. The control group was treated with 0.05% Tween 80. The insects were housed in an insect chamber with corn leaves, maintained at 28 °C, 70% relative humidity, and a 16L:8D light–dark cycle. Mortality was recorded daily, and dead insects were removed. The locust cadavers were maintained under humid conditions for 10 days to monitor conidial formation. Each treatment was repeated three times, and the entire experiment was conducted in triplicate.

### 4.5. Determining Chitinase and Lipase Activity in Fungi

A conidial suspension of *M. rileyi* at 1 × 10^7^ conidia/mL was incubated at 28 °C, and chitinase and lipase activities were measured on the fifth day. Chitinase and lipase activity was quantified using assay kits from Boxbio Science (Beijing, China). Chitinase hydrolyzes chitin to produce N-acetylglucosamine (NAG), which co-heats with a base to produce an intermediate compound that can be colorimetrically reacted with p-dimethylaminobenzaldehyde. Lipase-catalyzed hydrolysis of oil esters to free fatty acids and determination of the rate of fatty acid production was carried out using the copper soap method. Both enzymes were characterized by measuring the change in absorbance value. Total enzymatic activity (U/mg protein extract) was determined in three protein samples from each strain. Each experiment was repeated three times.

### 4.6. Nontargeted Metabolomics Analysis of Hemolymph of L. migratoria

A conidial suspension of *M. rileyi* PPDB201006 and SZCY201010 at a concentration of 1 × 10^7^ conidia/mL was injected into *L. migratoria*. On the fourth day post-infection, 200 μL of hemolymph was collected, frozen, and stored before being sent to Qingdao Biopu Biotechnology Co., Ltd. (Qingdao, China) for non-targeted metabolomics analysis. Each sample was processed in six replicates. The extracted samples were analyzed using an UPLC-ESI-MS/MS system (UPLC: Waters Acquity I-Class PLUS; MS: Applied Biosystems QTRAP 6500+). The analytical conditions were as follows: UPLC column, Waters HSS-T3 (1.8 µm, 2.1 mm × 100 mm). The mobile phase consisted of solvent A (pure water with 0.1% formic acid and 5 mM ammonium acetate) and solvent B (acetonitrile with 0.1% formic acid).

### 4.7. Fungal RNA Extraction and RT-qPCR

Fungal conidial suspensions of strains PPDB201006 and SZCY201010 at equal concentrations were inoculated onto SMAY plates and incubated at 25 °C for three days. Total RNA was extracted from 0.5 g of mycelium following a previously established method [37]. Reverse transcription into cDNA was performed using the PrimeScript^®^ RT kit (TaKaRa, Dalian, China). RT-qPCR primers were designed using the NCBI database, and each cDNA sample was diluted 20-fold as a template for real-time quantitative PCR (qRT-PCR) analysis. The primers used for qRT-PCR are listed in Table 1.

### 4.8. Data Analysis

One-way analysis of variance (ANOVA) was performed using SPSS 25.0 to analyze *M. rileyi* bioassays on *L. migratoria* as well as fungal growth and virulence measurements. Means were compared between fungal strains using Tukey’s Honestly Significant Difference Test (Tukey’s HSD). All statistical analyses were conducted using GraphPad Prism 9.5.0 at different significance levels.

## 5. Conclusions

Our results show that PPDB201006 is significantly superior to SZCY201010 in terms of its ability to utilize various carbon and nitrogen sources, as well as its enzyme activity and virulence against locusts. This study provides important insights into how *M. rileyi* utilizes carbon and nitrogen sources to increase its virulence against *L. migratoria*. The findings emphasize the potential of manipulating fungal metabolism to improve the effectiveness of entomopathogenic fungi as biological control agents.

## Figures and Tables

**Table 1:** Primer sequences of real-time fluorescent quantitative PCR.

Primer Name	Primer Sequence (5′-3′)	Accession Number	Purpose
*MrVib1*	F: TTCTTATCGGAGATGCCCGC R: TCGTTGGCCTCAGTAACCAC	OAA45487.1	RT-qPCR
*MrNmrA*	F: GAAGTTCCAGCACCTGGCTA R: CCGTTGGACATGCACTTGGA	TWU77448.1	RT-qPCR
*MrNRR*	F: CAGCAACTGCCAAACCAACA R: TCGGGCGGTTATGTCTGATG	OAA42774.1	RT-qPCR
*MrAreB*	F: AGAGTCGCAACCGGGTAAAG R: TCGACACCAGTTACGTCAGC	OAA47622.1	RT-qPCR
*MrMAK*	F: GCTTCGGCAGTGTTGTTCTG R: TGATGGCCACAACTGAACCG	OAA44248.1	RT-qPCR
*MrPpc*	F: AGAACGTTGTCTTCGACCCC R: CATCGGGTGTTCTCGGTCAA	OAA50610.1	RT-qPCR
*MrMae*	F: ATGGTCGAGGTCCATCCTCA R: ACGTTGTCTGAAGTCGTCCC	OAA47740.1	RT-qPCR
*ACTIN*	F: CTTTTAATCGGCGCACGGAG R: CGAAGCTTGGCGCTATTGTC	OAA40333.1	RT-qPCR

## Data Availability

All data are available in the main text or the Supplementary Materials.

## Supplementary Materials

The following supporting information can be downloaded at: https://www.mdpi.com/article/10.3390/ijms26094156/s1.
