# Supplementary material for: The Virulence of Metarhizium rileyi to Locusta migratoria Is Determined by the Ability of the Fungus to Respond to Carbon and Nitrogen Sources
Source: Int J Mol Sci. 2025 Apr 27;26(9):4156. doi: 10.3390/ijms26094156 (PMC12071437; doi:10.3390/ijms26094156)

Supplementary Figure S1. Phylogenetic analysis of strains PPDB201006 and SZCY201010 based on EF-1α sequences. In the phylogenetic tree constructed using the Neighbor-Joining method, branches with bootstrap support values greater than 50% are labeled based on 1000 bootstrap replicates.

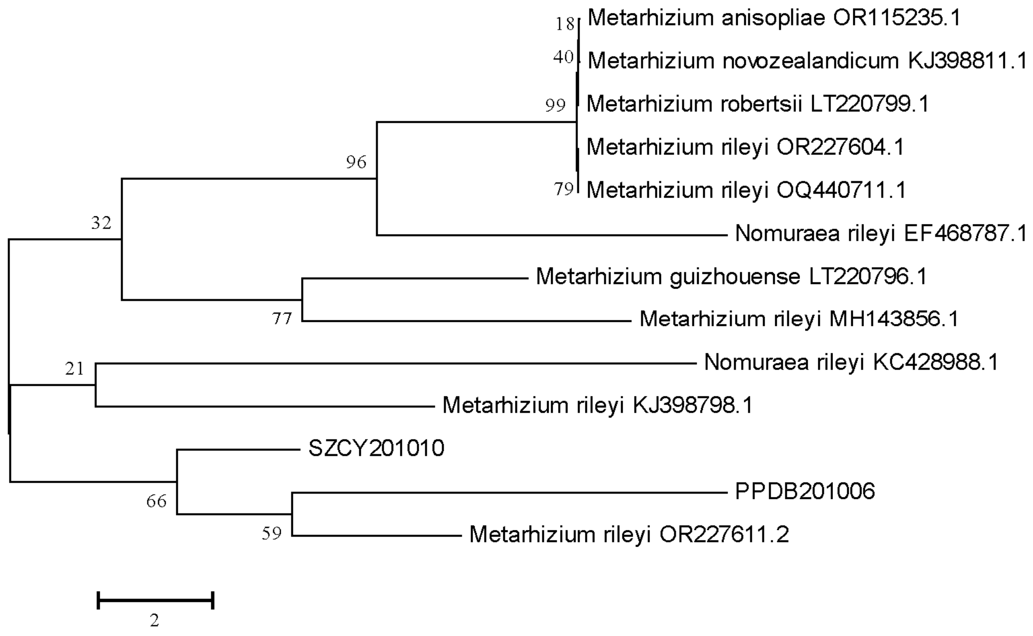

Supplement: Supplementary file 1 [file ijms-26-04156-s001.zip › Supplementary Figure.pdf]
